# Precision Medicine and the future of Cardiovascular Diseases: A Clinically Oriented Comprehensive Review

**DOI:** 10.3390/jcm12051799

**Published:** 2023-02-23

**Authors:** Yashendra Sethi, Neil Patel, Nirja Kaka, Oroshay Kaiwan, Jill Kar, Arsalan Moinuddin, Ashish Goel, Hitesh Chopra, Simona Cavalu

**Affiliations:** 1 PearResearch, Dehradun 248001, India; 2 Department of Medicine, Government Doon Medical College, HNB Uttarakhand Medical Education University, Dehradun 248001, India; 3 Department of Medicine, GMERS Medical College, Himmatnagar 383001, India; 4 Department of Medicine, Northeast Ohio Medical University, Rootstown, OH 44272, USA; 5 Department of Medicine, Lady Hardinge Medical College, New Delhi 110001, India; 6 Vascular Health Researcher, School of Sports and Exercise, University of Gloucestershire, Cheltenham GL50 4AZ, UK; 7 Chitkara College of Pharmacy, Chitkara University, Punjab 140401, India; 8 Faculty of Medicine and Pharmacy, University of Oradea, P-ta 1 Decembrie 10, 410087 Oradea, Romania

**Keywords:** precision medicine, cardiology, precision cardiology, hypertension, heart failure, myocardial infarction

## Abstract

Cardiac diseases form the lion’s share of the global disease burden, owing to the paradigm shift to non-infectious diseases from infectious ones. The prevalence of CVDs has nearly doubled, increasing from 271 million in 1990 to 523 million in 2019. Additionally, the global trend for the years lived with disability has doubled, increasing from 17.7 million to 34.4 million over the same period. The advent of precision medicine in cardiology has ignited new possibilities for individually personalized, integrative, and patient-centric approaches to disease prevention and treatment, incorporating the standard clinical data with advanced “omics”. These data help with the phenotypically adjudicated individualization of treatment. The major objective of this review was to compile the evolving clinically relevant tools of precision medicine that can help with the evidence-based precise individualized management of cardiac diseases with the highest DALY. The field of cardiology is evolving to provide targeted therapy, which is crafted as per the “omics”, involving genomics, transcriptomics, epigenomics, proteomics, metabolomics, and microbiomics, for deep phenotyping. Research for individualizing therapy in heart diseases with the highest DALY has helped identify novel genes, biomarkers, proteins, and technologies to aid early diagnosis and treatment. Precision medicine has helped in targeted management, allowing early diagnosis, timely precise intervention, and exposure to minimal side effects. Despite these great impacts, overcoming the barriers to implementing precision medicine requires addressing the economic, cultural, technical, and socio-political issues. Precision medicine is proposed to be the future of cardiovascular medicine and holds the potential for a more efficient and personalized approach to the management of cardiovascular diseases, contrary to the standardized blanket approach.

## 1. Introduction

Over the past three decades, cardiovascular diseases (CVDs) have preponderated global disease burden––93% prevalence, 54% mortality, and 60% in disability-adjusted life years (DALY) [1,2,3]. This is further exacerbated by disparities in the inter- and intra-continental disease burden, costing USD 216 billion and USD 147 billion annually for healthcare and productivity loss, respectively [4,5,6].

The rectitude of medicine has always emphasized treating the patient rather than the disease. Today’s medicine is honing itself to be more precise and patient-centric. Precision medicine is an innovative clinical approach that uses individual genomic, environmental, and lifestyle information to guide medical management. It has already revolutionized oncology [7]; CVDs form their current epicenter owing to their heterogeneity and multi-causality, which leads to altered responses to treatment for each patient. The long-old treatment principles are succored by technological evolution in the “omics”—genomics, transcriptomics, epigenomics, metabolomics, proteomics, and microbiomics— which, together, help frame the position for future medicine [8]. “Omics” is aided by advanced “big data” analysis, which has helped in the development of in-depth clinical, biological, and molecular phenotyping, promoting better-integrated healthcare with early diagnosis, enhanced risk stratification, and disease management with the least possible side effects [9].

Most CVDs stem from a complex interplay of modifiable and non-modifiable factors which aggravate the set “omic” predisposition. In contemporary cardiology, most diagnostic criteria and therapeutic approaches rely on population-based studies, with less focus on approaches tailored to individualize patient treatment [10]. As such, a comprehensive analysis of phenotypes and the “omics” can help cluster patient groups sheathing disparities, simultaneously reinforcing patient-centric clinical care. Further, it will enhance patient quality of life (QOL) and help reduce complications via novel biomarkers, improved AI-assisted diagnostics, targeted therapeutics, and appropriate long-term risk assessment [11].

With technological advancements in data science and machine learning, the applicability of precision medicine in CVDs seems within reach, especially with the significant evolving literature in the pipeline over the past decade. As such, we aim to: 1. pool evolving clinically relevant information on precision medicine in cardiology, and 2. provide a comprehensive synthesis of the relevant literature to date. Thus, this will help with the evidence-based precise management of cardiac diseases and identification of possible challenges.

## 2. Precision Medicine and Cardiology

**Figure 1:**
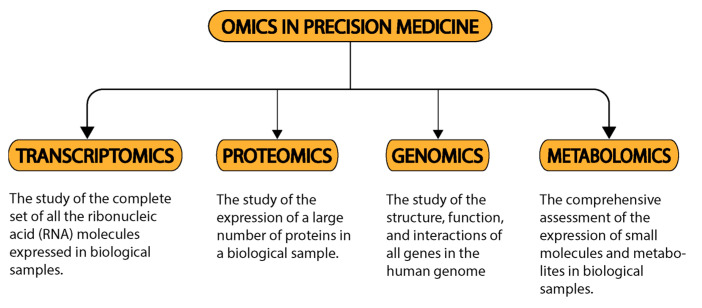
The OMICS in precision medicine.

**Figure 2:**
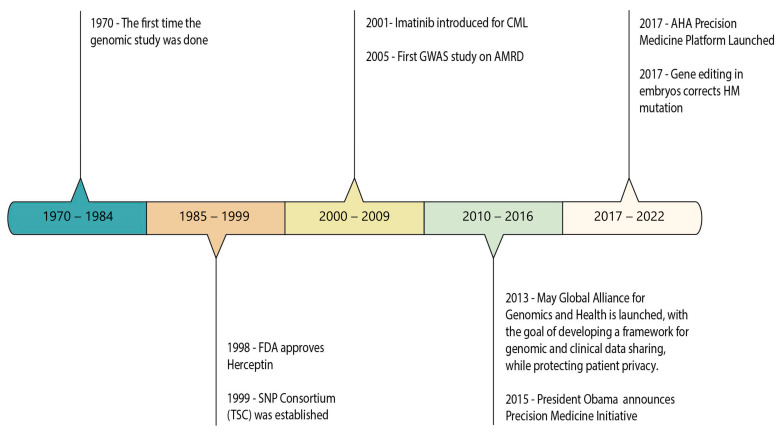
Timeline of the evolution of precision medicine in cardiology.

The advent of precision medicine has the potential to revolutionize the future of cardiovascular disease (CVD) healthcare via its application through “omics” in cardiology (Figure 1). It empowers a physician to treat cardiac diseases on an individual basis—based on the patient’s unique profile. Recent times have seen a growing body of literature underlining the application of precision medicine in cardiology. Table 1 presents a compilation underlining the clinical significance of all “reviews” published over the past decade regarding the same, while Table 2, Table 3 and Table 4 present an omic-stratified and disease-specific compilation of the literature for myocardial infarction, hypertension, and heart failure, respectively. As such, precision medicine in cardiology promises to improve health and revolutionize the management previously manifested in oncology. The evolution of precision medicine in cardiology has been remarkable (Figure 2). Its applicability can have the best impact if enacted on the diseases with the highest impact (associated with the highest DALY), these include—myocardial infarction, hypertension, and heart failure.

### 2.1. Myocardial Infarction

Myocardial infarction (MI) is the leading cause of death globally—16% of total deaths. Its pathogenesis is peculiar in terms of its heterogeneous causality and largely varied genetic predisposition. MI is a critical medical emergency, true to its scientific adage “Time is equal to Myocardium”. An opportune diagnosis with sensitive markers, optimal intervention, and the prevention of complications and recurrence is extremely consequential. Precision medicine may find its applications in all these areas (Table 2) and may guide research and drug development to add to the pharmacotherapeutic armamentarium for this disease [29,30].

**Table 2:** A compilation of the literature underlining the role of precision medicine in myocardial infarction.

S.N.	Authors	Year	Study Design	Clinically Relevant Findings	Comments	References
PROTEOMICS						
1	Felicita Andreotti et al.	2021	Literature Review	Biological sex (sex chromosomes, hormones, body size, social behavior, etc) affects the incidence of Chronic Coronary Syndrome (CCS).	Gender affects the incidence of CCS.	[31]
2	Zhanpeng Wen., et al.	2021	Scientific Report	Telmisartan-doped co-assembly nanofibers (TDCNfs).	Telmisartan-doped co-assembly nanofibers (TDCNfs) synergistically counter-regulate RAS and can be used in treatment post-MI.	[32]
3	Gaozan Tong., et al.	2020	Scientific Report	Basic fibroblast growth factor (bFGF).	The activation of NrF2 by bFGF lowers oxidative stress. This can significantly lessen the apoptosis of cardiomyocytes and the size of the infarcts brought on by MI, thereby lessening the damage to the heart.	[33]
4	Apurva Shrivastava., et al.	2020	Scientific Report	High-sensitive troponin, miRNAs, natriuretic peptides, CRP, and metabolites.	Deep phenotyping data, blood biobank, cardiac stress-MRI test, and identified candidate biomarkers (such as miRNAs, troponin, CRP, etc.) will be used to derive a biomarker specifically for ischemia.	[34]
5.	Filippo Crea., et al.	2019	Review	Myeloperoxidase and hyaluronidase-2.	Potential biomarkers that can non-invasively detect plaque erosion in ACS patients include myeloperoxidase and hyaluronidase-2.	[35]
6.	Rocco A Montone., et al.	2018	Review	- Radiographic biomarker: Unstable plaques; - Interruption of treatment with Ca antagonist during follow-up leads to a worse outcome.	A patient-specific treatment plan for MINOCA requires determining the cause. Some diagnostic tools for this purpose include the electrocardiogram (ECG), cardiac enzymes, echocardiography, coronary angiography, left ventricular angiography, coronary vasomotion, and intravascular imaging techniques.	[36]
7.	Kenneth Mangion., et al.	2017	Literature Review	Biomechanical parameters of LV function.	The biomechanical aspects of LV function, such as contractility, stiffness, strain, and stress, which are related to LV pump performance and, consequently, prognosis, can be learned through computational modeling.	[37]
8	Laura Pasea., et al.	2017	Scientific Report	BMI, WBC, haemoglobin, creatinine, and cholesterol ratio.	When deciding whether to continue DAPT therapy (in patients 1 year after acute MI), prognostic factors such as demographics, behavior, cardiovascular history, non-cardiovascular history, biomarkers, and medications should be taken into account.	[38]
9	Emeline Goretti., et al.	2014	Review	miRNAs.	miRNAs can be used to diagnose acute MI, stratify MI risk, and other medical conditions. After an MI, circulating miRNA levels have some prognostic value, and BNP’s diagnostic value is enhanced.	[39]
10	Elena Kaschina., et al.	2014	Review	AT2-receptor can be used post-MI for cardiac protection.	AT2R has a protective role in the heart after MI, leading to improved cardiovascular health. The protective function includes improvement of LV contractility, protection from early LV dilatation, and an antifibrotic effect under certain conditions.	[40]
11	Akinyemi Oni-Orisan., et al.	2014	Review	Epoxyeicosatrienoic acids (EETs).	Epoxyeicosatrienoic acids (EETs) possess cardioprotective effects as they elicit anti-inflammatory, vasodilatory, fibrinolytic, and anti-apoptotic effects. They can be used as a treatment for acute MI.	[41]
12	Nisha Arenja., et al.	2013	Scientific Report	Infarct size, LVEF Independent predictors of silent MI are: history of diabetes and CAD.	Better diagnostic capability is provided by imaging methods such as myocardial perfusion single-photon emission computed tomography or cardiac magnetic resonance. Infarct size, left ventricular ejection fraction, a history of diabetes, CAD, and other factors are independent predictors of silent MI.	[42]
GENOMICS						
13	Naveen Pereira., et al.	2020	RCT	CYP2C19 LOF mutation (or CYP2C19 LOF alleles carriers).	A genotype (CYP2C19 LOF mutation)-selected P2Y12 inhibitor instead of traditional clopidogrel shows no difference in ischemic events, thereby suggesting the incorporation of precision medicine earlier in the course of PCI.	[43]
14	Daniel Claassens., et al.	2019	RCT	CYP2C19 mutation (or CYP2C19 LOF alleles carriers).	After PCI, genotype (CYP2C19)-guided P2Y12 inhibitor selection was superior to standard treatment in terms of thrombotic events and had less bleeding events.	[44]
15	Robert A Scott., et al.	2016	RCT	Six genes (CNR2, DPP4, GLP1R, SLC5A1, HTR2C, MCHR1) that encode therapeutic targets in development of type 2 diabetes or obesity.	Six genes (CNR2, DPP4, GLP1R, SLC5A1, HTR2C, MCHR1) encode the therapeutic targets in development of type 2 diabetes or obesity. Therefore, these genes can be used to develop drugs with more efficacy and minimal side effects.	[45]
METABOLOMICS						
16	Gaetano Tanzilli., et al.	2021	RCT	Glutathione.	Institution of early and prolonged infusion of glutathione in a ST-segment elevation MI patients having undergone PCI can reduce the inflammatory response, thereby protecting the myocardial cells and improving cardiac remodeling.	[46]
17	Bicciré F., et al.	2021	Observational study	Serum albumin level.	Less than normal serum albumin levels were predictive of complications in STEMI patients.	[47]
18	Sayanti Bhattacharya., et al.	2014	Case-Control Study	Branched-chain amino acid metabolites.	BCAA and related metabolites are associated with atherosclerosis.	[48]
19	Svati Shah., et al.	2012	Case-Control Study	Medium-chain acylcarnitines, short-chain dicarboxylacylcarnitines, long-chain dicarboxylacylcarnitines, long-chain acylcarnitines, short-chain acylcarnitines, medium-chain acylcarnitines, ketone-related, cholesterol, lipids, fatty acids, glucose, branched-chain amino acids and related catabolites, amino acids.	Branch-chain amino acids, medium-chain acylcarnitines, short-chain and long-chain dicarboxylacylcarnitines, and fatty acids were all independently linked to mortality. Fatty acids, on the other hand, were predictors of MI/death, as were short-chain, long-chain, and dicarboxylacylcarnitines.	[49]

#### 2.1.1. Metabolomics

The association between an individual’s metabolic profile and MI has been explored a lot recently. Svati Shah et al. demonstrated an independent association between peripheral blood metabolites and the presence of CAD (coronary artery disease). They showed that simple metabolites such as factor 4 (branched-chain amino acid metabolites) and factor 9 (urea cycle metabolites) can help diagnose CAD [48,50]. Many new metabolites have recently been associated with CAD, including but not limited to: medium-chain acylcarnitines, short-chain dicarboxylacylcarnitines, long-chain dicarboxylacylcarnitines, long-chain acylcarnitines, short-chain acylcarnitines, medium-chain acylcarnitines, ketone related, cholesterol, lipids, fatty acids, glucose, and branched-chain amino acids [49]. Another recent observational study by Tanzilli G et al. found that low serum albumin levels are associated with adverse events in STEMI patients [46]. Uchino Y et al. and Ijichi C et al. showed that supplementing patients with BCAAs (branch-chain amino acids) showed increased serum albumin levels [51,52]. The combination of one or more newer metabolic markers can aid the diagnosis for a certain subset of patients, personalizing their care and increasing the sensitivity of MI detection.

Metabolomics can improve treatment options in addition to its predictive and diagnostic capabilities. Currently, the mainstay of acute coronary syndrome treatment is revascularization via emergency percutaneous coronary intervention (PCI). Tanzilli G et al. proposed ways to improve PCI by using early and prolonged glutathione infusions to blunt the inflammatory response via a chain of processes: 1. reduced NOX2 activation; 2. hsCRP generation; 3. TNF- levels; 4. cTpT release; 5. reduced neutrophil generation to protect myocardial cells; and 6. prevention of aberrant cardiac remodeling, allowing better left ventricular size and function post-PCI [46].

#### 2.1.2. Genomics

Advances in genomics are helping the knowledge gained to be incorporated into the treatment and diagnosis of MI. An RCT by Scott R et al. discovered six genes (CNR2, DPP4, GLP1R, SLC5A1, HTR2C, MCHR1) that could be potentially used to develop drugs to treat type 2 diabetes or obesity without incremental CVD risk [45]. At present, post-PCI, dual antiplatelet therapy (DAPT) is initiated to reduce the risk of thrombosis or MI. DAPT usually consists of aspirin and clopidogrel. However, patients with loss-of-function CYP2C19 mutation have elevated chances of ischemic events if treated with standard DAPT therapy [44]. Pereira N et al. showed that the genotype CYP2C19-guided selection of P2Y12 inhibitor was superior to standard treatment concerning thrombotic events and resulted in a lower incidence of bleeding. They also proposed that a genotype-guided P2Y12 inhibitor such as ticagrelor should be selected for such patients [43].

#### 2.1.3. Biomarkers

The development of biomarkers in precision medicine has been astounding and is still evolving. Mangion K et al. suggested the use of computational modeling to provide precise diagnostic care to patients and improve individual risk prediction. Computed biomechanical parameters such as contractility, stiffness, myofilament kinetics, strain, and stress provide information about LV function, thus determining the extent of cardiac damage and future prognosis [37]. Crea F et al. established biomarkers such as myeloperoxidase and hyaluronidase-2 to identify plaque erosion among ACS patients [35]. The CD44v6 splicing variant of the hyaluronan receptor was significantly higher in patients with plaque erosion than in plaque fissures, which can help detect silent myocardial infarctions. Adding to the evidence of the benefit of therapy individualization, Pasea L et al. concluded that the decision to prolong DAPT therapy should be assessed individually for each patient [38]. For assessment, prognostic models should look at demographics and behavior, cardiovascular history, non-cardiovascular history, biomarkers, and drugs. Tong G et al. explored the use of basic fibroblast growth factor (bFGF) in myocardial ischemia injury [33]. Oxidative stress plays a major role in myocardial injury, and bFGF can reduce oxidative stress by promoting the activation of NrF2 via the Akt/GSK3b/Fyn pathway, which reduces cardiomyocytes apoptosis and thus the infarct size to a larger extent, ultimately alleviating the heart injury. More studies are being conducted to study the cardioprotective effect of bFGF, which is certainly a novel preventive tool to treat MI. Further, Oni-Orisan A et al. found that epoxyeicosatrienoic acid (EET) elicits potent anti-inflammatory, vasodilatory, anti-apoptotic, pro-angiogenic, fibrinolytic, and smooth muscle cell anti-migratory effects with-in the cardiovascular system [41], and thus can be used in the treatment of acute MI. Wen Z et al. investigated the application of telmisartan-doped co-assembly nanofibers (TDCNfs), dual-ligand supramolecular nanofibers that synergistically counter-regulate RAS through targeted delivery, and presented it as an option for combined therapy against cardiac deterioration post-MI [32]. It reduces apoptosis, alleviates inflammatory response, and inhibits fibrosis to potentially mitigate post-MI outcomes [32]. Aside from MI, the treatment for myocardial infarction with no obstructive coronary arteries (MINOCA) also requires an investigation of cause to determine the patient-specific treatment [36]. Collectively, precision medicine helps to individualize the therapy by blending various diagnostic modalities such as ECG, cardiac enzymes, other biomarkers, echocardiography, coronary angiography, coronary vasomotion, and intravascular imaging techniques.

### 2.2. Hypertension

Hypertension (HTN) is a major modifiable risk factor for cardiovascular morbidity and mortality. Globally, it forms a major share of NCD (Non-communicable diseases); about 1.28 billion adults aged 30–79 years have HTN [53]. Due to its insidious onset, the majority of HTN cases remain unidentified and run a silent but catastrophic course. Early detection and optimal control can considerably reduce the cardiovascular burden associated with it. The pathophysiology of hypertension includes an interplay between genetic, physiological, biochemical, and environmental factors, which vary amongst individuals [54]. Precision medicine in hypertension can specifically identify patient subgroups with distinct disease causation mechanisms and their differential responses to diverse antihypertensive treatments. A compilation of recent evidence for the application of PM in hypertension is listed in Table 3.

**Table 3:** A compilation of the literature underlining role of precision medicine in hypertension.

S.N.	Authors	Year	Study Design	Clinically Relevant Findings	Comments	References
METABOLOMICS						
1	Phil Chowienczyk et al.	2014–2023	Ongoing RCT	Pharmaco-metabolomics of antihypertensive drug response.	AIM HY study is underway to investigate biochemical pathways, pharmaco-metabolomics, and pharmacogenomics in antihypertensive drug response which can further be used for precision therapy in hypertension.	[55]
2	Diana M Rydberg et al.	2018	Cross-sectional study	Sex in antihypertensive drug response.	More adverse events (ADE) were seen in women compared to most antihypertensive drugs, however, aldosterone antagonists showed an increased incidence of ADE in men.	[56]
3	Yan Gong et al.	2016	Meta-Analysis	Obesity and ancestry in response to beta blockers.	In terms of ethnic diversity, diuretics and CCBs work better than ACEIs in African Americans due to the genetic variations in the RAAS. Obesity also influences antihypertensive drug response. Obese patients report better response to beta blockers due to their enhanced sympathetic activity, causing increased cardiac output.	[57]
4	Yidan Zhao et al.	2014	Experimental Study	Dicarboxylic acid (hexadecanedioate) as a causal pathway for hypertension.	Metabolomic profiling study on pulmonary hypertension supported a novel mechanism for blood pressure regulation that involved dicarboxylic acid (hexadecanedioate). It disrupted beta-oxidation and increased omega-oxidation pertaining to its novel putative role in both pulmonary and systemic hypertension. Identification of this metabolomic pathway potentiates further research in precision medicine for hypertension.	[58]
5	Lizzy M Brewster et al.	2013	Systematic Review	African ancestry in antihypertensive drug response.	African Americans are prone to severe hypertension due to higher vascular contractility and salt-holding capacity. Thus, ethnicity and ancestry provide a valuable marker, and a predictor of blood pressure lowering response to antihypertensives for devising individual treatment options is imperative.	[59]
6	William R Wikoff et al.	2013	RCT	Atenolol-induced changes in metabolome help understand the differential response to beta-blockers.	Metabolomic analysis of hypertension helps understand the disease as it is closely knit with the alteration of metabolic pathways. Atenolol-induced changes in the metabolome have been shown to depend on race and genotype, and this may explain the differential metabolomics of response to β-blockers in terms of blood pressure control.	[60]
GENOMICS						
7	Lakshmanan Loganathan et al.	2020	Literature Review	Identification of deleterious SNPs and target proteins in rational drug design through computational screening of mutagenesis.	The identification of deleterious SNPs involved in RAAS signaling genes that play an associative role with hypertension could contribute to antihypertensive drug discovery and diagnosis. Moreover, Cytochrome P (CYP) polymorphisms vouch for individual and population differences in drug tolerance.	[61]
8	Ian M. Kronish et al.	2019	Pilot Study	-	Personalized trial with antihypertensive treatment carried out with seven patients. The study found that patients were able to choose a particular antihypertensive drug at the end of the study period based on personal preference through daily blood pressure monitoring and were able to decide which treatment was personally more beneficial to them in lowering their blood pressure.	[62]
9	Boon-Peng Hoh et al.	2019	Literature Review	Ancestral alleles in salt homeostasis and identification of “Intermediate Phenotypes” for hypertension through GWAS.	Natural selection and evolution have a role in blood pressure variability due to varying frequencies of ancestral alleles that regulated salt homeostasis in humans. In a search carried out in the GWAS catalog in 2018, seven candidate genes with an established pathophysiological mechanism pertaining to hypertension were replicated in GWAS, namely ACE1, ACE2, ADRB1, ADRB2, MME, CACNA2D2, and UMOD. This implies that genetic variants concerning hypertension are comparatively more common (>1% in a population), with negligible natural selection in the past populations.	[63]
10	Yoshihiro Kokubo et al.	2019	Literature Review	Gene–environment and gene–lifestyle interactions in hypertension treatment.	Hypertension management must incorporate gene–environment interactions in addition to lifestyle components to prevent and treat hypertension. Gene–obesity, gene–physical activity, gene–sodium, gene–alcohol, gene–smoking, and gene–healthy-diet interactions contribute to the choice of treatment for hypertension as it is a multifactorial disease.	[64]
11	Evangelos Evangelou et al.	2017	Meta-Analysis	Polygenic risk score and genetic risk score, drug target genetic loci identified for drug development.	Application of polygenic risk score and genetic risk score in estimation of hypertension manifestation and drug response. It has been reported that five loci (PKD2L1, SLC12A2, CACNA1C, CACNB4, and CA7) are drug targets for hypertension medications. More than 1000 BP-determining loci have now been identified and drug-target genes are expected to expand in the future.	[65]
12	David L. Mattson et al.	2017	Editorial	Functional genomics in disease-relevant tissues.	Taking into account only DNA sequence for precision medicine in hypertension is an incomplete approach because hypertension is also influenced by environmental factors, which pose a challenge to quantify and identify. Functional genomics should be carried out in the disease-relevant tissues to contribute to precision medicine for hypertension.	[66]
13	Surendran P. et al.	2016	Meta-Analysis	Allelic variations in hypertension.	Identified 31 novel hypertension-associated genetic regions that include three unidentified missense variants in RBM47, COL21A1, and RRAS. Various associations were found in A2ML1 and ENPEP. These allelic variations lay the foundation for new drug targets for clinical interventions and management in precision hypertension.	[67]
14	Wenzheng Zhang	2015	Editorial	Modified sodium channel epithelial 1α subunit (SCNN1A).	Epigenomics has helped to define groups of hypertensive patients who respond superiorly to aldosterone-based therapies. It targets the epigenetically modified sodium channel epithelial 1α subunit (SCNN1A) in a specific group of hypertensive individuals.	[68]
15	Timo P Hiltunen et al.	2015	RCT	TET2, CSMD1 response to thiazide diuretic.	Identified TET2 and CSMD1 as loci linked to systolic blood pressure lowering response to hydrochlorothiazides.	[69]
16	Matteo Trudu et al.	2014	Cohort	UMOD gene polymorphisms in differential response to loop diuretics in hypertensive patients.	UMOD polymorphisms have been linked to a better BP response to loop diuretics in essential hypertension patients. “High” UMOD group has better hypertension control to loop diuretics while “low” UMOD group has a poorer response to loop diuretics.	[70]
17	Anne M Muskalla et al.	2014	Observational Study	G-protein receptor kinase 4 (GRK4) polymorphism response to antihypertensives.	GRK4 polymorphisms R65L, A142V, and A486V were analyzed, and it was found that homozygote double variants of 65 L and 142 V required more antihypertensive therapy than did homozygous single variants or heterozygous carriers to reach the optimal mean arterial BP reading.	[71]
18	Hye Duck Choi et al.	2013	Meta-Analysis	ADD1 and ACE I/D polymorphisms’ role in HCT response.	ADD1 polymorphism Gly460Trp: The carriers of the Gly/Gly genotype had an enhanced response to hydrochlorothiazide compared to carriers of the Trp/Trp genotype.	[72]
19	J D Duarte et al.	2012	Original Study	PRKCA and GNAS-EDN3 in response to thiazide.	On administration of thiazide diuretics, PRKCA (rs16960228) allele carriers had better hypertension control than GG homozygote patients. Additionally, GNAS–EDN3 rs2273359 G allele carriers showed a better blood pressure response than CC homozygote patients.	[73]
TRANSCRIPTOMICS						
20.	Brian G Bazzell et al.	2018	Experimental Study	The RNA-sequencing of urinary vesicles (extracellular) on molecular modification in the mineralocorticoid receptor activation showed changes in the mRNA found in urine supernatant. This may contribute to better-tailored pharmacological treatment in mineralocorticoid signaling disorders such as resistant hypertension. Furthermore, it may help in the noninvasive identification of new putative biomarkers for cardiovascular and renal diseases and also predict drug responses.	Measurement of mRNA transcripts in the urine as a marker for mineralocorticoid receptor activation can help predict the response to mineralocorticoid receptor antagonists in hypertension.	[74]

#### 2.2.1. Hypertension Pharmacogenomics

Genetic polymorphisms and response to diuretics: Current evidence identifies maximum target polymorphisms in response to thiazide diuretics. As opposed to the findings of the GenHAT study, the homozygous carriers of ACE I/D polymorphisms, ACE II, showed a small reduction in blood pressure response to hydrochlorothiazide, compared to homozygous carriers of ACE DD alleles [72,75]. Another study reported the response to thiazide diuretic in African Americans with SNP rs7297610 CC located on chromosome 12q15 [76]. Other genetic association studies showed that PRKCAA allele carriers had a better blood pressure response than GG homozygote patients. Another study targeting the UMOD gene polymorphism showed differential BP response to loop diuretics in hypertensives. They divided patients into two groups: 1. UMOD group (AA genotype), which showed a good response to loop diuretics, and 2. “Low” UMOD group, which exhibited a lower BP response to loop diuretics [70].

Genetic polymorphisms and response to other antihypertensives: Blood pressure regulation in humans involves at least 70 genes and presents with a complex set of individual differences. The human beta (1)-adrenergic receptor (ADRB1) has two common functional polymorphisms (Ser49Gly and Gly389Arg), which are associated with varied responses to metoprolol in essential hypertension. Furthermore, 49Ser389Arg/49Ser389Arg and 49Ser389Arg/49Gly389Arg polymorphisms have been seen as good responders, whilst 49Ser389Gly/49Gly389Arg and 49Ser389Gly/49Ser389Gly polymorphisms have been seen to be non-responders [77]. ADRB1 polymorphisms further revealed that C allele homozygotes showed a better response to metoprolol than G allele carriers [78]. Another study reported a possible link between nephrin (NPNS1) gene variants and a good response to the angiotensin receptor antagonist, losartan, in hypertensive patients [79]. Other less commonly studied genetic polymorphisms include GRK4 polymorphisms, namely R65L, A142V, and A486V. Studies have revealed that homozygote double variants of 65 L and 142 V require more aggressive antihypertensive therapy than the homozygous single variants or heterozygous carriers to achieve a target mean arterial blood pressure [71].

Another study aimed at finding rare and common variants associated with hypertension identified 31 novel genetic regions—rare missense variants in RBM47, COL21A1, and RRAS [67]. These allelic variations can lay the foundation for newer drug targets. Furthermore, by using polygenic risk score (based on the total number of genetic loci required to be assessed to estimate the risk of developing a disease), five potential loci (PKD2L1, SLC12A2, CACNA1C, CACNB4, and CA7) have been reported as novel therapeutic targets for hypertensive therapy. More than 1000 hypertension-associated loci have now been identified, with drug-target genes expected to expand in the future [65]. Loganathan et al. identified other hypertension-associated loci and SNPs (Single Nucleotide Polymorphisms) such as RAAS signaling and Cytochrome P (CYP) genes, which govern individual and population differences in drug tolerance [61]. In 2018, the GWAS catalog found seven candidate genes with an established pathophysiological role in hypertension, namely ACE1, ACE2, ADRB1, ADRB2, MME, CACNA2D2, and UMOD [63].

Recently, newer drugs such as Rostafuroxin have disrupted the binding of mutant alpha-adducin and the ouabain-activated Na-K pump with the Src-SH2 domain, in rats as well as in human cell cultures, posing as potential antihypertensives [80]. Evidence also suggests that riboflavin, a co-factor for MTHFR, also has an antihypertensive effect via the MTHFR 677TT genotype-specific mechanism [81]. Another potential choice is aldosterone, which targets the epigenetically modified sodium channel epithelial 1α subunit (SCNN1A). It hypomethylates the histone protein (H3) at lysine 79 (H3K79) at subregions of the promoter in a subgroup of hypertensives [68].

#### 2.2.2. Hypertension Metabolomics

The effect of metabolomic factors in hypertension is another limb to study for the precision medicine domain. The most commonly identified metabolomic factors include sex, gender, race, and plasma renin activity; their response to antihypertensives was studied. A growing body of literature found that hypertensive women have lower plasma renin activity as opposed to men, and thus were more responsive to diuretics and Calcium Channel Blockers (CCB) as compared to angiotensin-converting enzyme inhibitors (ACEI) and beta blockers [82]. Another study comparing renin profiling-guided (plasma renin activity) treatment to clinical judgment in uncontrolled hypertensive patients showed equal or better hypertension control while using the renin profile-guided treatment approach [83]. Adverse drug events following antihypertensive medications were more common in females, though a notable exception was aldosterone antagonists [56]. It has been shown that African Americans respond to diuretics and CCBs better than ACEIs, probably due to their RAAS genes and increased plasma level in conjunction with suppressed plasma renin activity. These factors are hypothesized to cause variations in drug responses, Additionally, the African race is predisposed to severe hypertension, courtesy of their enhanced vascular contractility and salt-retaining capacity [59]. Furthermore, a raised sympathetic tone amongst obese patients responds better to beta blockers [57]. Ongoing clinical trials are investigating biochemical pathways, pharmaco-metabolomics, and pharmacogenomics in antihypertensive drug responses.

#### 2.2.3. Resistant Hypertension

A recent experimental study by Bazzell et al., on hypertension transcriptomics, measured mRNA transcripts in the human urine supernatant to detect mineralocorticoid receptor activation and predict its response to mineralocorticoid receptor antagonists in hypertensive patients. The results of the RNA sequencing of urine extracellular vesicles match those of the human kidney. Alterations in mRNA in urine supernatant were associated with changes in human endocrine signaling (MR activation). These findings can aid in individualizing pharmaceutical therapy in patients with mineralocorticoid signaling abnormalities, such as resistant hypertension. These findings could be utilized to noninvasively discover possible indicators of abnormal renal and cardiorenal physiology [74]. In light of the current evidence available, it can be concluded that what we know about the pharmacogenomics of hypertension is only the tip of the iceberg, and finding more precise targets and therapies is imperative.

### 2.3. Heart Failure

Heart failure (HF) is one of the most challenging cardiovascular disorders to manage. Despite recent advances in symptom management and the possibility of halting disease progression, the structural and functional impairment associated with HF is irreversible. It is a disorder with heterogenous causality and a strong genetic predisposition. Thus, risk assessment, prevention, and early screening are key in its management. Precision medicine poses to fill the existing gap in preventive medical management and risk stratification (Table 4).

**Table 4:** A compilation of the literature underlining role of precision medicine in heart failure.

S.N.	Authors	Year	Study Design	Clinically Relevant Findings	Comments	References
PROTEOMICS						
1	Leanne Dumeny et al.	2021	RCT	NR3C2 rs5522 G allele.	NR3C2, which codes for the spironolactone target protein, and CYP11B2, which is implicated in aldosterone synthesis, were linked to improved spironolactone responsiveness in diastolic HF patients.	[84]
2	Shah S et al.	2020	Meta-Analysis	KLHL3 and SYNPOL2–AGAP5.	Causative of HF.	[49]
3	Maurer, M. S et al.	2018	RCT	Gene-encoding autosomal dominant Val122Ile variant.	Tafamidis reduced death and hospitalizations associated with cardiovascular events in patients with transthyretin-associated cardiomyopathy.	[85]
4	Dominguez, F et al.	2018	Review	BLC2-associated athanogene 3 (BAG3).	Loci KLHL3 and SYNPOL2–AGAP5 are implicated in of HF, and also BAG3 and CDKN1A are associated with LV systolic dysfunction.	[86]
GENOMICS						
5	Julio Núñez et al.	2021	Original Study	Plasma carbohydrate antigen 125 (CA125).	CA125 is a surrogate of fluid overload, hence potentially valuable for guiding decongestion therapy, and a CA125-guided diuretic strategy improved eGFR in patients with acute heart failure with renal dysfunction.	[87]
6	Pierpaolo Pellicori et al.	2020	RCT	Collagen type I C-terminal telopeptide (CITP) and galectin-3.	Spironolactone affects various pathways that contribute to HF progression.	[88]
7	Feng SD et al.	2017	Observational Study	Beta-endorphin (β-EP) and brain natriuretic peptide (BNP) plasma concentrations.	Patients with acute left heart failure or atrial fibrillation can be identified early with excellent specificity and sensitivity using β-endorphin and brain natriuretic peptide.	[89]
8	Chester L. Drum et al.	2017	Comparative Study	Plasma Thymosin Beta-4 (TB4).	Heart failure with preserved ejection fraction (HFpEF) in females is associated with an increase in plasma TB4, which independently predicts mortality	[90,91]
9	G Michael Felker et al.	2015	RCT	High-sensitivity cardiac troponin T (hs-cTnT).	High levels of hs-cTnT were found in those with AHF. An increase in hs-cTnT, whether at baseline or peak, is associated with poor outcomes, most notably cardiovascular mortality at 180 days.	
10	Zoltán Pozsonyi, et al.	2014	Clinical Trial	Copeptin.	Copeptin can be used to predict a 5-year all-cause mortality in patients with heart failure.	[92]
11	Glick D et al.	2013	Observational Study	cTnT and NT-proBNP.	Systolic dysfunction, incident HF, and CV death can all be predicted from the long-term trajectory of cardiac troponin T(cTnT) and N-terminal pro-brain natriuretic peptide(NT-proBNP) in older adults without HF.	[93]
12	Hanna K Gaggin et al.	2013	Clinical Trial	sST2.	Soluble suppression of tumorigenesis (sST2) measurement identifies patients with chronic heart failure in whom higher beta-blocker doses may be beneficial	[94]
13	Shah RV et al.	2012	Original Study	MR-proANP and MR-proANP and MR-proADM.	Both mid-regional pro-atrial natriuretic peptide (MR-proANP) and mid-regional pro-adrenomedullin (MR-proADM) are effective for estimating prognosis in acute decompensated heart failure (ADHF).	[50]
14	Sjoukje I Lok et al.	2012	Original Study	Growth differentiation factor 15.	Growth differentiation factor 15 (GDF) levels are increased in patients with HF and correlate with the extent of myocardial fibrosis, hence they are used as a biomarker for cardiac remodeling	[95]
15	Natalia Lopez-Andrès et al.	2012	RCT	Galectin-3 (Gal-3), N-terminal propeptides of type I and III procollagens (PINP and PIIINP), and matrix metalloproteinase 1 (MMP-1).	Increased galectin-3 (Gal-3) and N-terminal propeptide III procollagen (PIIINP) levels, as well as low (metallic metalloproteinase-1 (MMP-1) levels, have been linked to poor long-term cardiovascular outcomes.	[96]
METABOLOMICS						
16	Olivotto, I. et al.	2020	RCT	Selective allosteric inhibitor of cardiac myosin ATPase.	Patients with obstructive hypertrophic cardiomyopathy saw improvements in exercise capacity, LVOT blockage, NYHA functional class, and health status after treatment with mavacamten, a small molecule modulator of β-cardiac myosin	[97]
17	Hunter WG et al.	2016	Original Study	Medium- and long-chain acylcarnitines and ketone bodies.	Patients with HFpEF had higher levels of medium- and long-chain acylcarnitines and ketone bodies than those with HFrEF.	[98]
18	Du Z et al.	2014	Original Study	3-hydroxybutyrate, acetone, and succinate, were significantly elevated in patients with HFrEF.	Patients with HFrEF had higher levels of 3-hydroxybutyrate, acetone, and succinate, all of which are predictive of HF outcomes.	[99]
19	Wang Li et al.	2013	Original Study	Lactate, alanine, creatinine, proline, isoleucine, and leucine in plasma.	Levels of lactate, alanine, creatine, proline, isoleucine, and leucine in plasma were all greater in patients with ischemic HFrEF compared to healthy controls.	[100]
20	Desmoulin F et al.	2013	Original Study	Plasma lactate and total cholesterol.	Patients with acute decompensated HF who have a high ratio of plasma lactate to total cholesterol have a significantly higher risk of dying within 30 days (ADHF).	[101]
MICROBIOMICS						
21	W H Wilson Tang et al.	2014	Original Study	Trimethylamine N-oxide (TMAO).	Increased mortality risk over the long run was associated with elevated TMAO levels.	[102]

#### 2.3.1. Genomics

The relevance of common genetic variation in the susceptibility to and heritability of HF has recently been investigated through large-scale genome-wide techniques. F Dominguez found that DCM (dilated cardiomyopathy) caused by mutations in BAG3 has high penetrance in carriers >40 years of age and increases the risk of progressive heart failure [103]. Shah S et al. found that loci KLHL3 and SYNPOL2–AGAP5 are implicated in HF, and also BAG3 and CDKN1A are associated with LV systolic dysfunction [104]. Maurer, MS et al., documented that tafamidis reduced death and hospitalizations associated with cardiovascular events in patients with transthyretin-associated cardiomyopathy [85]. Dumeny et al. found that NR3C2, which codes the target protein of spironolactone, or CYP11B2, which is involved in aldosterone synthesis, was associated with better spironolactone response in diastolic HF patients [84].

#### 2.3.2. Proteomics

Glick D found that among older adults without HF with initially low cardiac troponin T(cTnT) and N-terminal pro-brain natriuretic peptide (NT-proBNP), the long-term trajectory of both biomarkers predicts systolic dysfunction, incident HF, and CV death [93]. Pozsonyi et al. defined that copeptin predicted 5-year all-cause mortality in heart failure patients. Drum et al., found that plasma TB4 is elevated in women with HFpEF, which predicts mortality independent of clinical risk factors and NT-proBNP in women with HF [92]. Feng SD et al. found that β-endorphin (β-EP) and brain natriuretic peptide BNP have both high specificity and sensitivity to detecting early acute left heart failure and atrial fibrillation in patients [89]. Pellicori et al., investigated the effects of spironolactone on the serum markers of collagen metabolism and cardiovascular structure and function in people at risk of developing HF, as well as the potential interactions with a marker of fibrogenic activity, galectin-3 [88]. G Michael Felker found that Hs-cTnT was elevated in the majority of acute heart failure (AHF) patients. Baseline, peak, and peak change hs-cTnT were associated with worse outcomes, mainly 180-day cardiovascular mortality [91]. Shah et al. found that MR-proANP seems accurate in diagnosing acute decompensated heart failure (ADHF), whilst both mid-regional pro-atrial natriuretic peptide (MR-proANP) and mid-regional pro-adrenomedullin (MR-proADM) acclimatize prognosis [50]. Sjoukje I Lok found that growth differentiation factor 15 (GDF) levels are increased in patients with HF and correlate with the extent of myocardial fibrosis, hence they are used as a biomarker for cardiac remodeling [95]. Natalia Lopez-Andrès et al. found that increased galectin-3 (Gal-3) and N-terminal propeptide III procollagen (PIIINP), and low metallic metalloproteinase-1 (MMP-1) are associated with adverse long-term heart failure outcomes [96]. Julio Núñez et al. suggested that CA125 is a surrogate of fluid overload, hence it is potentially valuable for guiding decongestion therapy, and a CA125-guided diuretic strategy improved eGFR in patients with acute heart failure with renal dysfunction [87]. Hanna K Gaggin et al. found that the soluble suppression of tumorigenesis (sST2) measurement identifies patients with chronic heart failure in whom higher beta-blocker doses may be beneficial [94].

#### 2.3.3. Metabolomics

Metabolomics is the study of tiny, organic compounds within metabolic pathways. With the improvement of technology, nuclear magnetic resonance, gas chromatography, and mass spectrometry have enabled the discovery and analysis of enormous databases of metabolites implicated in heart failure. The molecular pathways implicated in cardiac failure show that a metabolic transition occurs in the failing myocardium. Metabolic profiles of patients with systolic heart failure have been developed by examining patient serum and breath. These profiles can be used clinically for diagnosis and prognosis in this population [105]. Du Z et al. suggested that 3-hydroxybutyrate, acetone, and succinate were elevated in patients with HFrEF and can predict outcomes in patients with HF [99]. Additionally, Hunter WG et al. found that levels of metabolites in medium- and long-chain acylcarnitines and ketone bodies are higher in patients with HFpEF compared to patients with HfrEF [98]. Wang Li et al. suggested that patients with HFrEF with ischemic causes had higher levels of lactate, alanine, creatinine, proline, isoleucine, and leucine in plasma than healthy subjects [100]. Desmoulin F et al. found that an increased ratio of plasma lactate to total cholesterol is a significant predictor of 30-day mortality in patients with acute decompensated HF (ADHF) [101]. Ahmad T et al. found that increased circulating long-chain acylcarnitine metabolite levels in patients with chronic HF were associated with adverse clinical outcomes [106]. Treating patients with end-stage HF with long-term mechanical circulatory support resulted in significantly decreased circulating long-chain acylcarnitine levels, suggesting that levels of long-chain acylcarnitine can be used to prognosticate HF outcomes. Olivotto I et al. found that treatment with mavacamten, a small molecule modulator of β-cardiac myosin, improved exercise capacity, LVOT obstruction, NYHA functional class, and health status in patients with obstructive hypertrophic cardiomyopathy [97].

#### 2.3.4. Microbiomics

W H Wilson Tang et al. suggested that high trimethylamine N-oxide (TMAO) levels were observed in patients with HF, and elevated TMAO levels portended higher long-term mortality risk [102].

### 2.4. Precision Medicine and Aortic Diseases

The genetic basis of aortic diseases has long been known. Twenty percent of patients with thoracic aortic aneurysms and aortic dissection either have a family history or are associated with a syndrome such as Marfan syndrome, vascular Ehlers–Danlos syndrome, and Loeys–Dietz syndrome. Mutations of *ACTA2, MYLK*, and *MYH11* have been found to be associated with aortic disease [107]. Furthermore, genetic variants of genes *FBN1, SMAD3*, and *ACTA2* have also been shown to cause either syndromic or non-syndromic thoracic aortic aneurysm and dissection [108,109]. The recent updates have added evidence to support the role of pathogenic variants in *COL3A1, FBN1, MYH11, SMAD3, TGFB2, TGFBR1, TGFBR2, MYLK, LOX*, and *PRKG1,* predisposing to hereditary thoracic aortic disease. The above insight into the genes associated with aortic diseases can be added to the clinical database. Patients with clinical suspicion can be tested for genetic predisposition, and the results can be saved on their EHR (electronic health records) to help personalize their treatment [109,110].

## 3. Precision Cardiology and Artificial Intelligence

The evolution in tools of artificial intelligence (AI) and machine learning models has made it possible to incorporate multimodal and multidimensional omics, which promise enhanced diagnosis and treatment modalities for tomorrow. AI has the potential to usher in the next medical revolution and enhance precision medicine to stratify patients according to their phenotypic characteristics. The incorporation of AI into laboratory medicine and diagnostics can aid in better performing screening and confirmatory tests. AI can be used to generate insights by integrating powerful computing and analysis, thus allowing the system to think, learn and empower clinical decision-making with augmented intelligence [111]. The advances in artificial intelligence and data science have allowed for the automation of various critical thinking processes in medicine, including diagnosis, risk classification, and management, easing the workload of doctors and decreasing the possibility of making errors. It has many different uses in the workplace and the care of patients, from making doctors’ life easier to facilitating research. As a field that relies heavily on abstract reasoning and interpretation, cardiology is a natural fit for the introduction of AI. Clinical evaluation, imaging interpretation, diagnosis, prognosis, risk stratification, precision medicine, and therapy for various cardiac diseases have all benefited from the use of artificial intelligence. Clinical diagnostic accuracy, especially for pediatric cardiac diseases, has been bolstered by the application of neural networks and machine learning. AI has helped increase the diagnostic utility of imaging modalities such as cardiac magnetic resonance imaging, echocardiograms, computer tomography scans, and electrocardiograms. In pediatric cardiac surgeries, the introduction of AI-based prediction algorithms greatly improves post-operative outcomes and prognosis. Important clinical results can be used with suitable computer algorithms for risk classification and predicting treatment outcomes [112]. Although artificial intelligence has made medicine more precise and accurate, it still has a long way to go and has some serious limitations. The acceptability is hampered by difficulties such as a lack of adequate algorithms and their infancy, a lack of physician training, a concern of over-mechanization, and dread of missing the “human touch”. The generalizability of algorithms developed in standardized research environments employing high-quality data to heterogeneous real-world populations must be rigorously evaluated. Biases in training data, model overfitting, insufficient statistical correction for multiple testing, and limited accountability around the processes by which deep learning algorithms reach their outcome (“black box” systems) are just a few of the pitfalls of AI that can have serious consequences for the patients, and they necessitate careful consideration by researchers, clinicians, and regulatory bodies. Despite the challenges, we believe that AI will be the perfect assistant to clinicians in directing adult and pediatric cardiology in the future [112,113]. Table 5 underlines the clinical applicability of AI in Precision cardiology.

## 4. Cardiovascular Pharmacology and Precision Medicine

The clinical trials help us judge and predict drug outcomes with the best representative samples that evolve with phases of clinical trials, but the genetic variations, environmental factors, and idiosyncrasies are still strong enough to cause a fair number of adverse events and treatment failures.

The evolving approach of precision medicine advocates the individualization of therapy, directed by local regulations and guidelines based on novel markers and gene targets, which can help us define reasons for failure, thus evolving a better tailored patient-centric approach to curing diseases. Numerous examples of genetic diversity and DNA variants determining the response to a drug are already in common parlance and are continually used to modify treatment. For instance, warfarin, the most commonly prescribed anticoagulant medicine, has a narrow therapeutic window and has shown wide inter-individual variations [114,115]. Studies document around 10% to 50% variability in warfarin dose requirements per the patient genotype, notably SNPs in *CYP2C9* (CYP2C9*2, CYP2C9*3) and *VKORC1* (rs9923231) [115,116,117]. Additionally, genetic variants have been identified that show differences in the response to β-blockers (*ADRB1, ADRB2, GRK5, GRK4*); angiotensin-converting enzyme inhibitors (*ACE, AGTR1*); diuretics (*ADD1, NPPA, NEDD4L*); and Calcium Channel Blockers (*CACNB2, CACNA1C*) [26]. Further, clopidogrel, an antiplatelet medicine, is a P2Y12 inhibitor, and it shows great inter-individual variability–the clopidogrel non-responders [106,118,119]. The genetics purported behind this involve loss-of-function alleles in CYP2C19 (CYP2C19*2 and CYP2C19*3), which is thought to be associated with poor drug responsiveness, whilst the gain-of-function allele CYP2C19*17 is associated with increased bleeding risk [119,120]. Now, the COAG (Clarification of Optimal Anticoagulation through Genetics) trial and the EU-PACT (European Pharmacogenetics and Anticoagulant Therapy-Warfarin) have produced RCTs advocating genotype-guided drug dosing for warfarin, which prescribe dosing based on *CYP2C9* and *VKORC1* genotyping [121,122]. Precision medicine aided by pharmacogenomics and pharmacogenetic profiling poses to refine this area, bringing in next-generation care using enhanced phenotyping for disease stratification [123].

## 5. Precision Cardiology and the Omics

**Figure 3:**
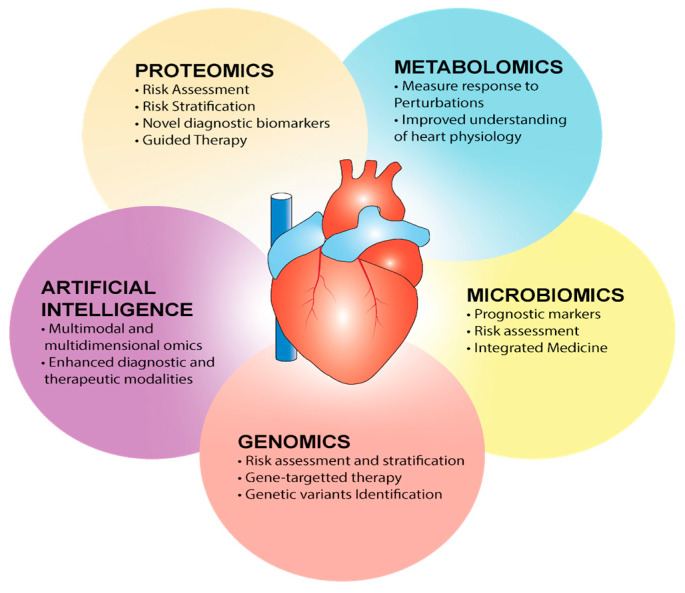
Domains of precision medicine in cardiology.

Cardiovascular research is increasingly a part of the vast, digital, data-driven world made possible by the plethora of molecular, physiological, and environmental data generated by a variety of “omics” technologies. Clinical research and practice will advance from focusing on the “typical patient” to gaining a more sophisticated understanding of specific individuals and populations [124]. With this review, we underline the key areas where the domains of precision medicine (Figure 3) can be implemented in cardiology diagnostics, stratification, therapeutics, and prognostics, and compare its novelty to the existing norm. Precision medicine empowers a physician to treat cardiac diseases individualistically, based on the patient’s unique genetic, metabolic, proteomic, or symptomatic profile. The strength of precision medicine lies in the synthesis and analysis of “data” that is rapidly changing from standard clinical, imaging, and laboratory testing to next-generation sequencing, metabolomics, and proteomic studies [8]. Modern-day cardiology is evolving to adopt new genetic, molecular, metabolic, and proteomic tools. In the case of myocardial infarction, newer biomarkers such as bFGF, hsCRP, hs Troponins, and miRNAs have emerged, which have great potential for detecting disease processes with more accuracy and at an earlier stage. Additionally, recent advances have shown that metabolites (such as acylcarnitines, fatty acids, BCAAs) are strong predictors of cardiovascular diseases and can be paired with standard metabolomics such as troponin and lipid levels to promptly predict the occurrence of MI/death in patients with heart disease. Similarly for heart failure, various markers such as 3-hydroxybutyrate, acetone, succinate 2-oxoglutarate, pseudouridine alanine, creatinine, proline, isoleucine, and leucine in plasma have shown usability for the prediction of outcome; various genes have also been identified that can serve the purpose of early risk stratification in the near future [125]. Despite its applicability challenges, genomics has contributed greatly to our understanding of the variability of disease processes, risk propensity, and response to treatment. Future advancements in genetic data generation and tools of application will enable its implementation in the routine management of common diseases. Next-generation sequencing and genome-wide association studies using a variety of computational biology technologies offer hope for improving the diagnosis and treatment of cardiovascular diseases. The mass spectrophotometric characterization of human cardiac proteins may expand the applicability of proteomics methods to CVD. Furthermore, transcriptomics approaches reveal novel information about gene expression, and metabolomics represents the tail end of multi-omics efforts to tackle CVDs early on. The “omics” can thus play a core role in the individualization of therapy in cardiac diseases [125].

## 6. Evolving Understanding of the Immune Cells and the Future of Precision Cardiology

The rupture of atherosclerotic plaques appears to be the leading primary cause of CVD. Atherosclerosis, the leading cause of CVD, is a chronic inflammatory condition in which immuno-competent cells in lesions produce primarily pro-inflammatory cytokines. One key target for atherogenic immune responses is heat shock proteins, with other mediators being: pro-inflammatory cytokines, chemokines, and lipid mediators [126].

The evidence has evolved significantly in this domain, highlighting role of immune cells in various cardiac diseases. To cite an example, in the pathophysiology of heart failure, regulatory T cells (Tregs) play a role in immunoregulation and tissue healing. Tregs help the heart by limiting excessive inflammatory response and encouraging stable scar formation in the early stages of cardiac damage. However, Treg phenotypes and functions are altered in chronic heart failure by these cells being mutated into antiangiogenic and profibrotic cells. In addition, tumour necrosis factor (TNF)- and tumour necrosis factor receptor (TNFR1) expression rises in HF-activated CD4+ T cells. Immunotherapy for heart failure is now conceivable because of advances in next-generation sequencing and gene editing technologies [127,128,129,130,131].

The majority of pharmaceutical therapies have focused on changing hemodynamics (lowering afterload, regulating blood pressure and volume) or cardiac myocyte function. However, significant contributions of the immune system to normal cardiac function and damage response have lately emerged as attractive research fields. Therapeutic approaches that harness the strength of immune cells have the potential to open up new therapeutic pathways for various cardiac diseases, and these form important targets for providing individualized therapy by exploiting the “omics” and tailoring therapy in line with the immune makeup of the patients [126,131,132].

## 7. Challenges to PM in Cardiology

Various experts question the applicability and accessibility of precision medicine, believing that it lacks a global impact on cardiovascular disease management and will merely serve a small group of patients in the developed world, relegating its role to a selected niche only. However, this concern seems implausible due to the limited literature attesting to the validity of this claim [133]. Another challenge is the dearth of acceptability and neophobia to the growing methods both by the providers and the recipients. Precision medicine was historically considered complex, expensive, and inaccessible to underserved populations.

Genomics has undoubtedly accelerated the discovery of mutations underlying cardiac diseases. Exploring genetic sequences, assembly, and the identification of genes is still evolving and seems to have a promising future, although the technology needed to translate this data into clinical interpretation and practice is still challenging. While major research work is focused on the exome (protein-coding DNA), another area of interest in present-day genetic sequencing is the non-protein-coding DNA and its impact on major clinical diseases, which are largely under-discovered. Moreover, the research/development of testing for genetic variants associated with the risk of developing a certain cardiac disease and its role in prevention is encouraging, but affordability and feasibility remain a concern even in developed countries [133,134].

Another challenge to PM in cardiology is the education and training of the stakeholders, including the providers and the general public [135,136]. Education must be aimed at training to use an integrated system approach, allowing healthcare providers and patients to be in congruency to accept and trust the new evolving techniques [136]. An added challenge is the apparent lack of available cohorts with relevant phenotypes to demonstrate statistically meaningful associations. Moreover, the absence of a replication cohort and differences in epigenomic patterns also make research difficult.

## 8. Future Perspectives and Conclusions

Precision medicine is the future of medicine and holds promise for the more efficient management of cardiovascular diseases, owing to their gradual onset and heterogeneous, multimorbid, and chronic nature. The pathogenesis of these diseases may begin decades before any ultimate disease manifestation. Therefore, the use of precisely targeted tools for diagnosis and personalized treatment can revolutionize management by allowing the prevention, early diagnosis, and tailored treatment of cardiovascular diseases. Precision medicine is still an evolving field and many of the technologies needed for its implementation are in nascent stages. Moreover, the research and data on precision medicine are limited because of the ethical, social, legal, and economic issues, which may have produced an unavoidable bias in this review as well. This review explored the literature on precision medicine in cardiology and tried to outline and summarize the most clinically relevant sections of the evolving field. As we evolve in our capacity and infrastructure to employ tools exploring the genomics, proteomics, and metabolomics of cardiovascular diseases, we stand to see a future where a more precise therapy tailored to the needs, demands and limitations of an individual patient would no longer be a dream but a responsibility. The future of cardiology is here; we need to assimilate, adapt and make it more accessible by educating the providers about the evolving field and making infrastructure more equitable to the public.

## Figures and Tables

**Table 1:** A compilation of important reviews underlining the role of precision medicine in cardiology.

S.N.	Authors	Year	Results	Clinical Significance	References
1	Guglielmo Gallone et al.	2022	The research focuses on how AI may bridge the gap between data-rich technologies and their deployment.	AI has the potential to use data-rich technologies in patient care, cardiovascular research, and health policy research.	[12]
2	Michael Simeon et al.	2021	It will be possible to use human pluripotent stem cells (hPSCs) in cardiovascular clinical care by developing isogenic hPSC cell lines as a control for hPSCs with disease-specific mutations and a large number of hPSC lines with gene mutations, for the in vitro modeling of human diseases with complex genotypes and phenotypes.	hPSCs with disease-specific mutations have application for use in cardiovascular clinical care.	[13]
3	Soni Savai Pullamsetti et al.	2021	Novel biomarkers can distinguish between left and right ventricular hypertrophy/failure.	Novel biomarkers can distinguish left ventricular hypertrophy/failure from right ventricular hypertrophy/failure, assess right ventricular disease severity, and potentially identify maladaptive changes in RV size, function, and architecture.	[14]
4	Farwah Iqbal et al.	2021	In both healthy and pathological cardiovascular tissues, scRNA-seq has enabled the characterization of heterogeneous cell subpopulations with distinct genetic profiles.	These can shed light on the pathological mechanisms underlying atherosclerosis and suggest new potential treatments for calcific aortic valve disease.	[15]
5	Concetta Schiano et al.	2021	Numerous ncRNAs, including miR-93, miR-340, miR-433, miR-765, CHROME, and large epigenetic changes in DNA methylation have been linked to atherogenesis in endothelial, smooth muscle, and macrophage cells.	In pro-inflammatory macrophages of the human carotid plaque, elevated HDAC9 was related to matrix metalloproteinase 1 (MMP1) and MMP2 production, while decreased HDAC9 was seen to promote resolution of inflammation and reverse cholesterol transfer, which may halt or reverse the disease process.	[16]
6	Christian Schulte et al.	2020	Emerging biomarkers—cardiac myosin binding protein C, SNPs, and non-coding RNAs.	Newer biomarkers, adding more specificity to the diagnosis.	[17]
7	Damien Gruson et al.	2020	In cardiovascular medicine, AI has shown promise as a tool to improve patient care and increase the efficiency of cardiologists.	The integration of AI and laboratory medicine can enable personalized care in cardiovascular medicine.	[18]
8	Valeria Visco et al.		New technologies in heart failure, atrial fibrillation, and cardiac rehabilitation offer a low-cost, non-invasive solution to long-term monitoring and management.	The availability of current devices with massive datasets offers a valuable tool for predicting the progression and outcome of various cardiovascular illnesses. Due to continuous monitoring, this new guided therapy can provide rapid and tailored treatment while also having a substantial psychological impact on patients.	[19]
9	Samuele Ambrosini et al.	2020	Building personalized maps of cardiovascular risk and designing individualized diagnostic and treatment approaches can be made possible by the integration of epigenetic and genetic data.	Genomics—customized diagnostic and therapeutic strategies.	[20]
10	Ziwei Pan et al.	2020	Induced pluripotent stem cells (iPSC)—functional evaluation of genes, patient risk stratification, testing of drugs, and personalized medicine.	With the help of this overview, readers should be better able to comprehend the value of iPSC-CM models, their various features, and their potential.	[21]
11	Danielle Menosi Gualandro et al.	2019	1. The interpretation of Hs-cTnT/I concentrations should always be performed quantitatively, and not in a binary fashion, as with pregnancy tests; 2. New hs-cTnT/I elevations show that “false elevations” from analytical issues are less likely than the “true elevations” due to an implicit or underestimated cardiac disorder such as heart failure causing myocardial injury.	The interpretation of Hs-cTnT/I concentrations should always be carried out within the context of the clinical presentation of the patient, using all other clinical information available and not in isolation.	[22]
12	Javier Rodríguez-Carrio et al.	2018	The CXCR4 pathway was associated with the effect of aging on Endothelial Progenitor Cells, whereas TNFα was found to be associated with hypertension. The response to dyslipidemia and diabetes-related traits was observed to be the activation of Akt/eNOS. The EPC dysfunction at the time of smoking is due to inflammation and oxidative stress.	Feasible biomarkers for risk stratification in personalized medicine schemes are suggested to be inflammatory and immune networks.	[23]
13	J. David Spence et al.	2018	Phenotype-based approach for HTN: 1. Regarding the Liddle phenotype (low renin/low aldosterone due to ENaC overactivity)—the specific therapy is amiloride; 2. In terms of the phenotype in primary aldosteronism—aldosterone antagonists; adrenalectomy; In terms of the renal phenotype—the renin/angiotensin system antagonists; renal revascularization.	Therapeutic approaches for hypertension can be best chosen based on specific phenotypes. The Liddle phenotype is more likely to be present in black hypertensives. They also tend to retain salt and water.	[24]
14	Perry V. Halushka et al.	2018	Evolving role of miRNA biomarkers as a diagnostic tool in diseases such as coronary artery vasculopathy, diabetic cardiomyopathy, aortic stenosis, and atrial fibrillation.	miRNA-based biomarkers can help as tools for specific early diagnosis.	[25]
15	Gemma Currie et al.	2018	It is suggested that the following steps should allow the translation of precision medicine into practical cardiology: (1) Make use of existing data; (2) Improve diagnostic tests; (3) Standardize phenotypes; (4) Techniques for the standardization and simplification of omics to support large-scale epidemiological studies; (5) Change the mindset in cardiovascular medicine toward molecular diagnostics; (6) Conduct stratified clinical trials.	Sex/gender have not yet been completely studied in precision medicine; nonetheless, the prospect of using molecular data to better properly manage men and women with cardiovascular disorders has been recognized.	[10]
16	M Zaiou et al.	2018	Genetic defects may be included in clinical practice since they may affect how common cardiovascular medicines such as aspirin, clopidogrel, warfarin, and statins interact with different individuals and influence how CVDs develop.	Knowledge of genomics can help the process of individualizing therapy.	[26]
17	Calum A. MacRae et al.	2016	The mainstay of modern cardiovascular therapeutics is small molecules such as antibodies against ANGPTL4 or Statins and PCSK9 inhibitors for lipid lowering. Genome-wide association studies (GWAS) have been successful in defining new loci leading to common diseases.	The potential for precision medicine-based therapeutic approach to cardiovascular disease care is immense, but so are the challenges for the same.	[27]
18	Toshio Nishikimi et al.	2013	Adrenomedullin (AM)—a potent vasodilatory peptide. It can also act as an autocrine and/or paracrine factor. Increased adrenomedullin is associated with the defense mechanism against further elevation of peripheral vascular resistance in cases of heart failure.	AM can be used in cardiovascular diseases for diagnosis and treatment.	[28]

**Table 5:** Clinical applicability of artificial intelligence in precision cardiology [11,111].

Clinical Applicability
1. Precision disease stratification
2. AI-aided diagnostics—probable diagnosis and risk stratification from imaging and investigations
3. Continuous remote monitoring and wearable devices
4. Telemedicine and remote diagnosis
5. Extension of Physician efficiency and efficacy
6. Integration of multi-omic data
7. Therapy selection
8. Database integration and reporting
9. Healthcare research and analysis
10. Patient education and information

## Data Availability

Not applicable.
